# Editorial: Solutions for organ transplantation: Xenotransplantation and interspecies chimaeras

**DOI:** 10.3389/fcell.2023.1194654

**Published:** 2023-04-11

**Authors:** Shoulong Deng, Dengke Pan, Yifan Dai, Jun Wu

**Affiliations:** ^1^ Institute of Laboratory Animal Sciences, Chinese Academy of Medical Sciences and Comparative Medicine Center, Peking Union Medical College, Beijing, China; ^2^ Sichuan Academy of Medical Sciences and Sichuan Provincial People’s Hospital, Chengdu, China; ^3^ Department of Medical Genetics, School of Basic Medical Science, Nanjing Medical University, Nanjing, China; ^4^ Department of Molecular Biology, University of Texas Southwestern Medical Center, Dallas, TX, United States

**Keywords:** xenotransplantation, blastocyst complementation, immune rejection, organoid, organ transplantation

Organ transplantation stands out as one of the most remarkable achievements of modern medicine, enabling the preservation of innumerable lives. In parallel, the advancement of human organ transplantation technology brings forth sanguine prospects for the management and cure of diverse pathologies. Nevertheless, the primary impediment in organ transplantation remains the dire dearth of donor organs. Current strategies such as donor donation, Xenotransplantation, organoid, 3D printing, and other modalities promise to alleviate this predicament. However, various obstacles still beset the preclinical and clinical application of these technologies. For instance, despite its vast potential, the successful realization of interspecies chimaeras has so far been achieved only in rats and mice. Reduced chimerism efficiency of human pluripotent stem cells in animal embryos could be attributed to suboptimal culture conditions, evolutionary divergence, and developmental incompatibility of pluripotent stem cells. There are still many problems to be solved in this field. It aims to delve into the advances in the physiology of organ transplantation, blastocyst complementation, strategies to enhance interspecies chimaeras’ efficacy, and ethical considerations about this field. Furthermore, the fabrication of distinct types of organoids and the post-transplantation immune rejection remain subjects of global interest. This Research Topic tries to present a comprehensive overview of the latest progress in the discipline of organ transplantation.

Immune rejection is a pivotal factor affecting the survival of transplanted organs. The suppression of immune rejection constitutes a crucial goal in the field of transplantation research. Although numerous researchers maintain that the adaptive immune system is the fundamental factor influencing transplantation rejection, the role of innate immunity in this process is increasingly being scrutinized. Zhang et al. present a review of how autophagy regulates these processes and propose potential targets for alleviating immune rejection. To begin with, several crucial autophagy-related proteins can interact directly with PRRs or the signaling molecules downstream of PRRs, such as ATG16L1-NOD1, ATG5/12-RIG-I, TRAF6-Beclin-1, and others. Consequently, autophagy plays a role in regulating innate immune signal transduction in organ transplantation. Moreover, autophagy regulates the inflammatory response and oxidative stress by directly degrading pro-inflammatory cytokines and oxidizing intermediates or targeting NLRP3, type II interferon, and PI3K signaling during multiple graft transplants. Furthermore, autophagy contributes to various forms of cell death, thereby influencing primary graft dysfunction. Non-etheless, under severe transplant stress, such as prolonged ischemia or severe reperfusion injury, autophagy invariably intensifies the inflammatory response, oxidative damage, and cell death. Despite the majority of studies still centring on observing autophagy activity during organ transplantation, the specific mechanisms underlying the interaction between autophagy and innate immunity remain poorly understood. Furthermore, organ transplantation is a complex process that elicits various forms of stress, such as mechanical injury, mucosal injury ischemia, hypoxia, hyperoxia, and bacterial translocation. However, most studies focus on only one of these factors while ignoring the combined effect of these stimuli on autophagy. Therefore, selecting an appropriate experimental model is crucial to comprehend the function of autophagy in graft transplants.

The use of immunosuppressive agents has been shown to greatly increase the incidence of malignancy. Overall, multiple studies have consistently demonstrated that organ transplant recipients have a significantly higher risk of developing malignancies than the general population. The risk of cancer in these patients is mainly affected by the type of transplant, which is determined by three factors: the use of immunosuppressive medications, underlying medical comorbidities, and end-stage organ disease. Shen et al. provide a summary of the utilization of high-throughput sequencing and organoids in the domain of organ transplantation, and the mutational patterns of cancer genomes, and present a novel research strategy for comprehending the mechanism of cancer following organ transplantation. Sequencing the genomes of *de novo* malignancies in post-transplant patients will continue to uncover cancer genes and mutational signatures that were previously unrecognized. Whole-genome sequencing, novel statistical methods, and innovative mathematical models will aid in answering these questions. By utilizing detailed clinical information, these newly discovered signatures can be combined with treatment and clinical responses to identify the driver genes for cancer development and to develop targeted drugs that will benefit patients. With the use of next-generation sequencing techniques, there will be a systematic analysis of the genome of *de novo* malignancies following organ transplantation, providing a comprehensive analytical perspective on the mutational processes of human cancer development soon.

The brain is the largest and most complex organ, with intricate neural activity. The brain’s power, particularly in humans, encompasses movement, sensation, vision, hearing, and more advanced brain functions such as consciousness and memory. The complexity of brain structure and function, particularly the distinctive functional division of the human brain, presents challenges to brain research. Chen et al. provide a comprehensive review of the establishment and development of brain organoids, encompassing the evolution of the technology. They also highlight the significant progress made in the exploration of brain development, neurological disease simulation, and drug screening, which has been made possible by brain organoids. Additionally, they analyze the possible future directions of the technology, including the potential to further advance studies of brain development and disease modelling, developing personalized medicine, and exploring new therapeutic targets. Due to ethical constraints and limitations in human brain tissue sources, researchers have traditionally employed animal models to investigate human brain development. The development of brain organoid technology not only overcomes the limitations of traditional 2D cell culture, which cannot replicate the complex structure of brain tissue and the *in vivo* microenvironment, and cannot reproduce the intricate phenotypes of neurological diseases, but also overcomes the shortcomings of lacking human-specific genetic characteristics, brain regions, and functions, along with the challenges of comprehensively simulating the development of the human brain and the limitations of disease occurrence and development. Brain organoids are a rapidly-emerging biological culture technology with immense research potential and application value in the study of human brain development, disease mechanisms, tissue replacement therapy, and drug screening. 

Whole organ generation *via* the blastocyst complementation approach holds great promise as a readily available resource for cellular therapies, and as a radical treatment option for most terminal diseases. However, whilst blastocyst complementation is emerging as a potential organ transplant option, challenges remain, such as organ size scalability, immune system incompatibilities, long-term maintenance, potential evolutionary distance, and uncovered mechanisms between donor and host cells. These challenges can be overcome by adopting a multifaceted approach, particularly by addressing knowledge gaps on the mechanisms of interspecies chimaera formation. Choe et al. and Sarmah et al. provide a summary of the history of interspecies chimerism in various animal models to offer insights into blastocyst complementation application and describe the challenges and prospects of utilizing blastocyst complementation for human organ generation. In addition, swine is thought to be a promising model for xenotransplantation. Utilizing gene editing technology primarily targeted towards genetic modification of the porcine vasculature, the first porcine cardiac xenotransplantation was performed in early 2022. These triumphs have further paved the way for the production of human-porcine chimeric organs. Through a gene deletion strategy and blastocyst complementation, human vasculature and skeletal muscle have been genetically engineered, and initial chimeric embryos have been assessed. Further studies are necessary to scrutinize the developmental progression of the human-porcine chimeric organs, the recipient animal model’s immunological response/tolerance, strategies aimed at enhancing interspecies chimaeras’ efficiency, and the comprehensive characterization of the human chimaeras. Collectively, these preliminary studies have generated immense enthusiasm and excitement focused on the prospects of curing end-stage diseases and democratizing organ transplantation using xenografts and exogenic chimeric organs.

In summary, this Research Topic has widened and deepened the understanding of solutions for organ transplantation. Here, we thank all the authors who contributed to this work a lot. Furthermore, their papers shed light on potential innovative therapeutic approaches to enhance public health safety and preserve human lives.

